# Physical Activity Habits and Well-Being among 6-Year-Old Children: The “Improving Umbrian Kids’ Healthy Lifestyle”, an Uncontrolled Pilot Study Project

**DOI:** 10.3390/ijerph17176067

**Published:** 2020-08-20

**Authors:** Roberto Pippi, Livia Buratta, Alessandro Germani, Carmine Giuseppe Fanelli, Claudia Mazzeschi

**Affiliations:** 1 Centro Universitario Ricerca Interdipartimentale Attività Motoria (C.U.R.I.A.MO.), Healthy Lifestyle Institute, University of Perugia, Via Giuseppe Bambagioni 19, 06126 Perugia, Italy; carmine.fanelli@unipg.it; 2 Department of Philosophy, Social, Human and Educational Sciences, University of Perugia, Piazza G. Ermini 1, 06123 Perugia, Italy; livia.buratta@studenti.unipg.it (L.B.); alessandro.germani@unipg.it (A.G.); claudia.mazzeschi@unipg.it (C.M.)

**Keywords:** healthy habits, school-based programs, multi-component assessment

## Abstract

There is evidence that promoting physical activity programs and decreasing sedentary behavior is a potential strategy for improving health-outcomes, peer relationships and social/emotional well-being in at-risk youth. The World Health Organization recommends enhancing physical education and school-based programs with multi-component and evidence-based assessment methodology. In Umbria (Italy) an uncontrolled pilot study project referred to as “Improving Umbrian kids’ healthy lifestyle” was implemented as a systemic school-based intervention directed at 6-year-old primary school children. The intervention applied a consolidated assessment methodology developed by the C.U.R.I.A.Mo. and Eurobis projects that inserted two hours per week of physical education activity into the school curriculum, structured and supervised by specialists with Exercise and Sport Science degrees, for eight months (from October to June) of the school year. We measured anthropometric values (BMI, waist circumference, waist-to-height ratio index) with objective tools. Moreover, we evaluated physical performance variables (speed, strength, and flexibility) using standard tests. Additionally, self-report measures (measured physical activity during the week, sedentary habits, and psychological well-being) were assessed using validated questionnaires. We observed a significant decrease in waist to height ratio, and improvements in physical performance values and self-report questionnaire measures. Our study suggests that the promotion of physical activity in the school setting is likely to result in physically, mentally, and psycho-socially healthier primary-school-age children.

## 1. Introduction

The World Health Organization (WHO) published a new global action plan on physical activity (PA) [1] to counteract a decrease in the time spent in PA and an increase in sedentary behaviors among children and adolescents that strongly predicts future adverse health outcomes [2]. Lack of exercise in childhood leads to an inactive lifestyle as adults [3]. The promotion of PA by children contributes to constructing competence and motor skills [4], fundamental in developing and maintaining adequate physical fitness in adolescence and adulthood [5]. Unhealthy habits (such as inactivity and low PA levels) that begin early in life seem to continue throughout life [6] and have become one of the most critical public health problems in the last century [7]. PA from an early age, in fact, can help to control weight and to prevent the risk of overweight, obesity and correlated physical diseases (cardiovascular risk, diabetes, colon and breast cancer [8]). Research has disagreed about the link between PA and weight at various developmental ages: studies suggest an association for adolescents but not for the youngest children [9], whereas other have not found any association for either adolescents or children [10]. Moreover, research studies have focused on the contribution of PA to the increase in the overall well-being of people throughout their lives. PA is associated with mental health, and cognitive and general psychological functioning both in children and youth [11]. Specifically, low PA levels are correlated with a prevalence of anxiety symptoms [12] and exercise was found to be effective in reducing depression [13,14]. PA may also improve children’s body image [15,16] and increase self-esteem [17]. Further, PA seems to have a positive influence on cognitive skills, and on the brain’s structure and function [18,19,20], particularly in memory [21] and attention [22].

Even though the WHO recommends a minimum of 60 min of moderate to vigorous PA daily between the ages of 5 and 17 years, many children in the world do not meet the recommended PA levels [23,24]. In Italy, the Surveillance System “OKkio alla salute” [25] of Italian National Institute of Public Health, who collect data on children’s weight, PA levels and lifestyle habits, showed that 23.5% of children engage in structured sports for no more than an hour a week, almost 40% of children dedicate more than one hour to structured sports activity two times a week, while only 2% spend more than one hour 5–7 days a week in structured sports (with a strong regional variability).

Since 2018, the WHO proposed 20 evidence-based policy actions on PA [1], recommended to enhance physical education and school-based programs. Considering that the teaching hours prescribed for physical education are significantly variable from one country to another and from one level of education to another [26], young people should increase physical education activity at school and should integrate it with a large amount of daily PA outside of school. To reach this goal, some evidence suggests that structured educational programs in the school setting, characterized by nutrition education and changes in eating habits and associated with increased PA [27,28], are strategic. There is also evidence that promoting PA programs and decreasing sedentary behavior is a potential strategy for improving peer relationships and social/emotional well-being in at-risk youth [29,30] as well as possibly protecting mental health in children and adolescents [31].

In this context, for the first time, Umbria (Italy) implemented the project “Improving Umbrian kids’ healthy lifestyle”, an intersectoral and systemic school-based intervention directed at 6-year-old children in primary school, using a consolidated assessment methodology of the C.U.R.I.A.Mo. and Eurobis projects [32,33]. The main aim of this project was to improve children’s lifestyle by intervening in some risk factors associated with overweight/obesity, with particular attention to physical activity, inserting, for the first time, two hours of physical education activity into the weekly school curriculum,. In an innovative way, lessons were structured and supervised by specialists with Exercise and Sport Science degrees.

This paper aims to present the effects of the school-based intervention on children’s anthropometric values (BMI, waist circumference, waist-to-height ratio index, measured using objective tools) and physical performance variables (speed, strength and flexibility, evaluated using standard tests), as well as on self-report measures (level of PA during the week, sedentary habits and psychological well-being) assessed using validated questionnaires. Furthermore, given that the intervention could not affect all children equally, another aim has been studied if, at the end of the intervention, there was a different change or improvement for boys and girls and for children of different BMI categories. The different weight status of the children may influence physical condition, motivational and emotional state and may affect their way of integrating in the intervention.

## 2. Materials and Methods

### 2.1. Procedure

The participants were collected through a convenience sampling of children attending the first level of elementary school from throughout the region of Umbria, and their parents, who took part in the “Improving Umbrian kids’ healthy lifestyle” project. In accordance with Italian school system protocols, each school was free to choose to participate in the project. Once a school had chosen to participate in the project, the following was provided:

- A short training period for school managers, teachers and specialists about the anthropometric and performance values, supervised by C.U.R.I.A.Mo. experienced health technicians.
- PA sessions (two hours per week) for all the children, included in the school curriculum by a special regional law [34], structured and supervised by specialists with Exercise and Sport Science degrees, based on Italian school Training Offer Plan (Piano Offerta Formativa (POF)) and educational activities within the motor-ludic strategy to promote the discovery of the feeling of fun through active games. Outside these two hours, the boys had no other physical activities scheduled during official school hours.
- Four educational meetings (three hours each session, one meeting every two months) about healthy nutritional education, short food supply chains for promoting local food, and obesity determinants. These meeting were addressed to children and their parents.

To evaluate the effect of the eight months of school-based intervention before (T0, in October) and after (T1, in June) the intervention, a multi-component, evidence-based methodology of assessment, using several standardized measures, was used. Anthropometric and performance variables for all participants were measured by specialists with Exercise and Sport Science degrees. In addition, children and their parents were invited to complete a general socio-demographic form and a validated self-report questionnaire to assess the level of PA during the week and the psychological well-being dimension. For this study, we selected only the children and parents who subscribed to the informed consent proposed.

The inclusion criteria were the presence of all data for anthropometric and performance measures and self-report questionnaires collected both before and after the intervention. The exclusion criteria were the absence of an adequate gym structure in school where the PA sessions were carried out, and the presence of any proven medical condition that would contraindicate the practice of PA.

The study was run in compliance with the guidelines laid down in the Declaration of Helsinki. The intervention followed the lifestyle approach and assessment methodology for children and adolescents of the C.U.R.I.A.Mo. Institute [32] approved by the local Ethics Committee, CEAS Umbria (HREC number 1/10/1633, 2010).

### 2.2. Participants

**Figure 1:**
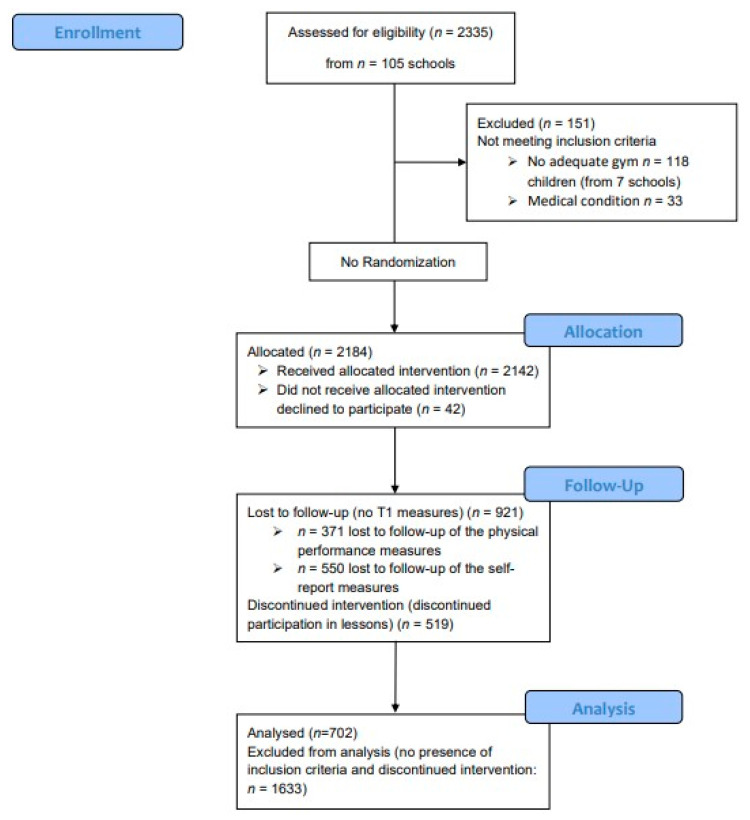
CONSORT Flow Diagram (adapted from CONSORT 2010 Flow Diagram).

The final sample of this study consisted of 702 Italian children (Figure 1) of 6 years of age, homogeneously distributed by gender (47.7% males, 52.3% females; Chi-square = 1.46; *p* > 0.05), and their parents. A total of 97.5% of these children was born in Italy. A total of 16% had at least one parent who was not born in Italy. In 92.9% of cases, the parents were married.

According to the classification of the World Health Organization (WHO) [35,36] children were grouped into three BMI categories: 69.2% were of normal weight (*n* = 486); 17.5% were overweight (*n* = 123); 13.2% were obese (*n* = 93). These percentages are in line with data from the Surveillance System of Italian National Institute of Public Health [25].

The families of these children have a middle-income level; the parents’ socio-economic status was measured by Socioeconomic status (SES, a construct that represents the social position of individuals or families relative to others [37,38]) was 33.00 (SD = 10.36). The mean age of mothers was 37.73 (SD = 4.90) and fathers was 41.06 (SD = 5.47). Referred mothers had a BMI in the normal weight category (<25: Mean = 22.78; SD = 3.75); whereas the fathers had a BMI at the limit of the overweight category (≥25: Mean = 25.80; SD = 3.43).

### 2.3. Measures

#### 2.3.1. Anthropometric Measures

All anthropometric values were assessed using standard techniques [39]. Height was determined using portable stadiometers; body weight was determined using a medical weighing scale; WC was measured with the individual in standing position at the end of expiration. The BMI was calculated according to the formula weight (kg)/(height (m)^2^, and used BMI cut-offs based on a WHO reference [40]. The waist-to-height ratio (WHtR) [41] was calculated according to the formula WC (cm)/height (cm) (defined as their waist circumference divided by their height), where a score < 0.50 indicates less visceral fat and low cardiovascular risk [42].

#### 2.3.2. Physical Performance Measures

Physical performance measures, SPEED, lower limb muscular strength (or STRENGTH) and flexibility from horizontal position (or FLEXIBILITY), were evaluated using age-specific tests [43,44,45]. SPEED was evaluated using the 30 m speed test during which children had to run the linear distance of 30 m, as quickly as possible. An exercise specialist ordered the start and recorded performance time (in seconds and hundredths) using a standard chronometer. STRENGTH values were estimated using the Sargent test, recorded as the difference (in centimeters) in the measures recorded after a vertical jump and the arm’s extended height, standing in front of a wall. FLEXIBILITY values were measured (in centimeters) using the Sit and reach test from a seated position.

#### 2.3.3. Self-Report Questionnaires Measures

A General Form (please see Figure S1 in Supplementary Materials) was used to collect socio-demographic information regarding the child and the family. It focused on family data such as socio-economic status (educational level and status of employment of the parents), parents’ marital status, demographic information and anthropometric measures as well as the lifestyle habits of the child (hours of sleep, time spent playing video games or watching TV, etc.).

PAQ-C Questionnaire was used to assess the child’s PA levels. It consisted of 10 questions (concerning PA in leisure time, in physical education lessons, during school days and during the weekend), resulting in expecting a score between 1 and 5 on a Likert 5-point scale. The PAQ-C total score (or PAQ-C) results from the arithmetic mean of the questions: 1 indicates poor PA level, while a value of 5 indicates high levels of PA practiced [46,47,48]. The PAQ-C has been indicated as a promising PA assessment in overweight and obese children [49] and appears to be an easy to use instrument for use in large epidemiological investigations [50]. This study used the Italian version that shows good internal consistency (α = 0.74).

Kid-Kindl is a self-report questionnaire that assesses the child’s quality of life (HRQoL). The KINDL consists of 24 items that evaluate, using a 5-point Likert scale from 1 (never) to 5 (all the time), six different domains of general well-being: physical well-being, emotional well-being, self-esteem, family, friends and everyday functioning (school). All scales are added together to generate a Total Score. High values (range 0–100) indicate a good HRQoL. This questionnaire shows good internal consistency (all subscales α > 0.60 and total score α > 0.80) This study used the Italian translation available on the official website [51].

### 2.4. Data Analysis

Descriptive analyses in terms of means, standard deviations or percentages were computed for each variable investigated before (T0) the intervention. The Independent *t*-test, Univariate Analysis of Variance (ANOVA) and Chi-square distribution were run to compare all variables between gender and across the BMI categories. The Repeat Measures Multivariate Analysis of Variance was used to compare all the assessment measures between T0 and T1, with gender and BMI categories as a between factor. The Chi-square was used to compare the sedentary habits during the week and during the weekend between T0 and T1 (after intervention) in the total sample and separately for each BMI category. *p* value < 0.05 was considered statistically significant in the analysis. Effect size was measured using partial eta-squares, in which small, medium, and large effects were 0.01, 0.06, and 0.14, respectively [52]. Statistical Analysis was performed using SPSS 18.0 (SPSS Inc. Released 2009. PASW Statistics for Windows, Version 18.0. Chicago, IL, USA).

### 2.5. Sample Size Calculation

A sample size of 673 achieves 90% power to detect a mean of paired differences of 0.2 on PAQ-C Questionnaire with an estimated standard deviation of differences of 1.6 and with a significance level (alpha) of 0.05 using a two-sided paired *t*-test.

The sample size calculation was performed using PASS Software (PASS 16 Power Analysis and Sample Size Software 2018. NCSS, LLC. Kaysville, UT, USA).

## 3. Results

### 3.1. Descriptive Analysis at the Baseline (T0)

#### 3.1.1. Anthropometric Measures

At T0 all anthropometric values (Table 1) showed mean equal between boys and girls, while the ANOVA highlighted differences across the BMI categories; in particular children, with obesity had a BMI, WC and WHtR greater than both children who were normal and overweight.

#### 3.1.2. Physical Performance Measures

In physical performance (Table 1), girls at T0 were more flexible than boys, while boys had faster and more lower limb muscular strength than girls. The ANOVA did not show any differences in the physical performances among BMI categories.

#### 3.1.3. Self-Report Questionnaires Measures

Before the intervention (Table 1), the mean of the PAQ-C showed medium levels of PA (2.62 ± 0.55): males seemed to show more activity than females during the week. The ANOVA and the Post-hoc analysis highlighted that the children who were of normal weight had higher levels of PA than children with obesity, with small effect size.

Regarding sedentary behavior, at T0 the sample showed a higher percentage of low inactivity levels both during the week and during the weekend. The Chi-square did not show any differences in the distribution between genders, while it showed a significant difference in the distribution among BMI categories, in particular a higher percentage of children with obesity reported more hours of inactivity during both the week and the weekend.

Regarding the quality of life perception, the sample at T0 reported high scores (>70) in all dimensions of well-being. The independent *t*-test and ANOVA did not highlight any significant differences between genders and across BMI categories.

### 3.2. Differences between before (T0) and after (T1) Intervention

#### 3.2.1. Anthropometric Measures

After the intervention (Table 2), all the samples did not show significant changes in BMI and WC, but significant interaction with BMI categories was shown with small effect size, In particular, the BMI significantly increased in the normal weight category (T0 = Mn 15.27, Sd 1.23; T1 = Mn 15.44, Sd 1.33; *p* < 0.001), while it decreased, but not significantly, in the overweight (T0 = Mn 17.83, Sd 0.48; T1 = Mn 17.82, Sd 1.17; *p* = 0.624) and obesity category (T0 = Mn 20.81, Sd 2.12; T1 = Mn 20.59, Sd 2.52; *p* = 0.184). Regarding the WC, it decreased only in children with overweight (with no statistical significance) at T1 (T0 = Mn 60.98, Sd 4.03; T1 = Mn 60.47, Sd 4.07; *p* = 0.108), differently in the normal weight (T0 = Mn 55.27, Sd 3.95; T1 = Mn 55.67, Sd 4.10; *p* = 0.005) and the obesity (T0 = Mn 67.49, Sd 6.19; T1 = Mn 67.76, Sd 6.19; *p* = 0.502) categories; the mean scores at T1 only significantly increased in the normal weight category.

Indeed, the total sample showed a significant decrease in WHtR, with interaction with BMI categories with small effect size, with a greater decrease in children with overweight (T0 = Mn 0.51, Sd 0.04; T1 = Mn 0.49, Sd 0.03; *p* < 0.001) and obesity (T0 = Mn 0.55, Sd 0.05; T1 = Mn 0.54, Sd 0.05; *p* = 0.003) than in children who were of normal weight (T0 = Mn 0.46, Sd 0.03; T1 = Mn 0.46, Sd 0.03; *p* < 0.001), but no with gender differences.

#### 3.2.2. Physical Performance Measures

The Repeated Measures Multivariate Analysis of Variance highlighted differences between T0 and T1 (Table 2) in all performance measures with an effect size between medium to large. No significant interaction with gender and BMI categories for any physical performance variable was observed. At T1, flexibility and strength significantly increased. Regarding speed performance, after the intervention, the children were significantly speedier than at baseline.

#### 3.2.3. Self-report Questionnaires Measures

After the intervention (Table 2), the children showed higher levels of PA than at baseline, with a medium effect size and no significant interaction with gender and BMI categories. Regarding sedentary behavior, as shown in Table 3, at T1 the Chi-square highlighted an increase in the percentage of the children in the low inactivity levels category, during the week but above all during the weekend, in the Total Sample. In particular, at the end of the intervention, during the week in the overweight category, 7% of the children moved from the category of greatest inactivity levels to the category with the lowest inactivity levels. Instead, during the weekend, the children who were of normal weight (4%) and with obesity (5%) decreased their inactivity levels.

Regarding the quality of life perception, some dimensions showed an increase after the intervention, with no significant interaction with gender and BMI categories. In particular, the Repeated Measures Multivariate Analysis of Variance highlighted an increase in physical, emotional and friendship well-being dimensions, with a small effect size.

## 4. Discussion

The aim of the present pilot study was to present the effects of “Improving Umbrian kids’ healthy lifestyle”, a school intervention project. Dedicating sufficient time to sport and PA at school, within the formal curriculum, can decisively help promote healthier lifestyles [26]. In fact, we observed an improvement in the anthropometric (WHtR decreased, *p* < 0.01), physical performance (increases in speed, strength, and flexibility, *p* < 0.01) and different lifestyle habits (improvement in PA level, *p* < 0.01) and well-being (social activities with friends, *p* = 0.001, and felt more accepted by their peer group, *p* = 0.028) variables.

In recent years, the WHO has reported a decrease in PA and an increase in sedentary behavior during childhood and adolescence. To cope with this, because children spend most of their daily time in school, the WHO suggested the implementation of physical education and school-based programs [53], recommending that they be evidence-based, multi-component and include assessment [1]. In Italy, the physical education teaching at school is not provided in all elementary classes (mainly due to the lack of adequate spaces, structures and gyms) and the practice of PA is left to the voluntary initiative of families who can decide independently whether to have their children practice some kind of sport, in extra-curricular hours. Moreover, depending on a school’s autonomy and staff resources, physical education teachers are generalist teachers, rarely graduated in Exercise and Sport Science degrees. The strength and the innovative aspect of the project in our study is the inclusion of two hours a week of mandatory PA in the official school timetable, with lessons of the school curriculum supervised by an Exercise and Sport Science degrees specifically formed for this project. This obtained encouraging results, such as increased levels of PA being practiced.

For these reasons, recent research has focused on the evaluation of multi-component clinical [45] and school-based actions [54], highlighting that programs have a greater impact if they involve the family [27]. Morano and coll. [54] showed that school programs may have sustainable benefits in reducing adiposity indicators and improving exercise adherence, physical fitness, and psychological well-being. For previously discussed reasons and to counteract unhealthy lifestyle habits in Italian children [25], particularly low PA level, we developed the school-based project “Improving Umbrian kids’ healthy lifestyle”.

In previous studies, standardized measures were not always used to assess school programs and PA levels. Measurement devices, time, and/or period, varied substantially across studies [55]. In fact, different methods are available for assessing PA levels and health-related PA measures among children and adolescents (e.g., energy expenditure, heart rate, subjective measures such as self-report, interviews, proxy-reports, and diaries [56]). In line with the WHO recommendation of evidence-based, monitored/assessed intervention [1], we adopted the C.U.R.I.A.Mo. model [32] and consolidated an assessment methodology, well verified in adults [57] and children and adolescents [45].

At baseline, our data showed that the community sample studied had a WHtR index mean, a simpler indicator of abdominal obesity that has greater practical advantages than traditional BMI and WC, as suggested by Ashwell et al. [58], in the normal weight category, both for boys and girls. Data showed low visceral fat and low cardiovascular risk.

Despite physical performance being considered an important indicator of health in young people [59], to the best of our knowledge there is a scarcity of physical performance reference standards for children, particularly in Italy. According to cut off values of sex- and age-specific fitness for pre-pubertal European children [60], we observed in our study, before the intervention, that boys performed better than girls in speed and muscular strength, while girls performed better than boys in flexibility. Fiori and coll. [61] found a discrepancy in physical fitness between BMI categories in children older than 6 years old. Our findings, differently to a recent study [61], did not show differences in physical performance among the BMI categories: children who were of normal weight were fast, flexible and strong, as well as children who were overweight or with obesity.

Regarding PA during the week, the sample showed medium levels (PAQ-C score >2.6) with boys showing higher levels of PA than girls, in accordance with Gobbi et al. [50]. Regarding the differences across BMI categories, our findings were in line with previous studies that highlighted that children with obesity were less physically active and had lower physical fitness than normal weight children [62].

The entire sample showed a higher percentage of low levels of inactivity both during the week and on the weekend, without differences between males and females. Children of all BMI categories were less active on weekends than on weekdays, as suggested by Soric and coll. [63] Furthermore, in line with the literature [64], it seems that children with obesity were the ones who spend the most sitting time than other children.

Finally, as to quality of life, the children, both boys and girls, showed high levels of well-being in all domains. All scores were higher than mean scores of the normative sample [51], as is to be expected in a community sample. Differently to the findings findings of a review [65], that showed that most of the studies suggested that the dimensions of quality of life are affected in children with overweight and with obesity, our results did not differ across the BMI categories, as has been shown in a few studies, probably because it was conducted in a general population [66,67] and not in a clinical sample, as the most of the other ones were.

At the end of the school-based intervention, our findings highlighted an improvement in several analyzed variables; the changes in most of these ones were not different either between boys and girls or between BMI categories.

As others have found [68], we did not observe significant differences between before and after intervention in BMI, as well as in WC, mean values, in all sample. We can hypothesize that this was due to the normal BMI values recorded before the intervention. However, BMI showed different changes among the BMI categories, with a statically significant change in normal weight group. Moreover, the important rule that child’s growth in BMI that can change due to alterations in height, bone density, muscle, fat or water should be underlined [69,70]. Finally, the duration of the interventions is an important variable in the reduction in the anthropometric variables, as shown by Adom et al. [68] in their review. Our study, lasting eight months, did not show statistically significant changes in BMI and WC, and future studies of longer duration are needed to clarify these aspects. Instead, in line with other authors [71], our data showed a statistically significant decrease in the WHtR index, decreasing even more the cardiovascular risk. WHtR index was the only variable that showed a different decrease among the BMI categories, with a greater decrease in children with overweight and obesity. At T1, the WHtR index of children with overweight decreased below the clinical cut-off, reducing the cardiovascular risks.

With regard to PA performance, it should be underlined that the expression of these abilities is related to other components that can cause the “effects” observed, such as the level of motor abilities [72] and individual changes in biological factors, such as growth and fitness level [24,70]. Our results are in line with those of Martinez Vizcaino et al., which observed significant improvements in muscular strength and velocity in boys and girls [73] and with those of Magnani et al., which observed improvements in strength values, as measured by the Sargent Test, in boys and girls of six years old [74].

The most recent Italian data of “OKkio alla salute”[25] showed that 41.2% of children (8 and 9 years old) spend more than two hours a day in front of a TV and/or playing video games/tablets/mobile phones (9.0% spent 5 or more hours in sedentary activities). In our project, we observed an improvement in PA levels both in boys and girls (Table 2), and significantly (*p* < *0*.001) decreased sedentary habits level (Table 3), during the week in overweight children and especially in children who were normal weight and children with obesity during the weekend.

It is well known that PA has a positive impact on mental health and quality of life domains in both children and adolescents [75,76], improving mood and several aspects of well-being including in the psychosocial dimension [77], the latter being very important at this time of life. In line with this evidence, our findings highlighted that at the end of the school-based intervention, the children reported a higher level of well-being in three different analyzed domains (physical, emotional and friendship). With regard to physical and emotional well-being, while the differences between T0 and T1 showed a low effect size, at the end of the first year of the project the children seemed to begin to perceive more energy, less physical discomfort, more vitality, less fear and less boredom compared to before. This perception may continue to increase and stabilize in the subsequent years of the project. A follow-up measurement could show these significant differences becoming more robust. The data showed the biggest difference between before and after the intervention, with a medium effect size in the friendship well-being domain. After the school-based intervention, children participated in more social activities together with their friends (*p* = 0.001) and felt more accepted (*p* = 0.028) by their peer group [Table 2]. Much research has shown that social interactions with peers and friends leading to perceived social support can influence the maintenance of PA at the time. Social support from peers seems to be a protective factor against a decline in activity levels among adolescents in later life [78,79,80]. It is important to underline that our result could be affected by repeated measurement effects and expected answering in self-report measurement. The change in PA and quality of life levels before and after the intervention did not differ across the BMI categories. 

No definitive conclusions can be based on this first edition of Project. In all the results, the changes found between the baseline evaluation and the end of the school year cannot be explained only by our intervention because the study lacked a control group and because the study is aimed only at 6-year-olds.

Further follow-up, controlled, research with relevant sample sizes and control groups is necessary to evaluate the long-term effect; it will be interesting to study how the “Improving Umbrian kids’ healthy lifestyle” working methodology can also be applied to groups of children of different ages and classes to those studied in this project.

## 5. Limitiations

This study has some limitations. First, our work lacked a control group, because it was not provided initially in the study design. Further, although originating from internationally validated tools, few outcomes were based on self-reported recall measures.

The tests chosen to assess physical performance are debatable. Performed during the physical education classes, the tests were chosen among those that were easy to perform in all the school environments, and only three of the several physical fitness tests suitable for the selected age group [81] were considered. Moreover, we did not use objective measurements (i.e., accelerometry) during PA sessions.

Finally, the age range (only six years old) and ethnicity of the study population could condition the generalizability of our results.

The large sample of children aged 6 that were evaluated, as well as the evidence-based methodology assessment using several standardized tests to assess physical measures and other variables, are strengths of this study. It is important to specify that the results presented in this paper are the first resulting/generated from the project, which could be implementated at other elementary school levels with subsequent follow-up evaluations.

## 6. Conclusions

This uncontrolled pilot study reports the first results of a school-based intervention, using an evidence-based approach and multicomponent assessment, aimed at improving six-year-old children’s PA level, inserting two hours/week of physical education supervised by specialists into the school curriculum. The findings of our study suggest that the promotion of more PA in the school setting is likely to result in physically, mentally, and psychosocially healthier children of primary school age.

## Figures and Tables

**Table 1:** Descriptive (Mn ± Sd or %) of all variables analyzed at T0.

	Gender				BMI Category					
	Boys	Girls	Boys vs. Girls		Normal Weight	Overweight	Obesity	NW vs. OW vs. OB		POST HOC
	Mn ± Sd	Mn ± Sd	*t*	*p*	Mn ± Sd	Mn ± Sd	Mn ± Sd	F _(__1.700)_, *p,* Partial η^2^		
Anthropometric measures										
BMI (kg/m^2^)	16.51 ± 2.28	16.41 ± 2.42	0.564	0.573	15.27 ± 1.23	17.86 ± 0.48	20.81 ± 2.12	797.05, <0.001, 0.69		1 < 2 < 3
WC (cm)	58.02 ± 5.91	57.75 ± 6.29	0.577	0.564	55.27 ± 3.95	60.98 ± 4.03	67.49 ± 6.19	349.47, <0.001, 0.50		1 < 2 < 3
WHtR (cm/cm)	0.48 ± 0.04	0.48 ± 0.05	−0.74	0.941	0.46 ± 0.03	0.51 ± 0.04	0.55 ± 0.05	259.18, <0.001, 0.426		1 < 2 < 3
Physical Performance measures										
SPEED (s)	7.83 ± 1.02	8.30 ± 1.18	−5.71	<0.001	8.03 ± 1.09	8.08 ± 1.15	8.32 ± 1.28.	2.55, 0.079, 0.007		
FLEXIBILITY (cm)	−0.70 ± 6.49	2.02 ± 6.69	−5.45	<0.001	0.81 ± 6.55	1.29 ± 6.77	−0.51 ± 7.49.	2.05, 0.130, 0.006		
STRENGTH (cm)	15.06 ± 5.79	13.95 ± 5.14	2.70	0.007	14.58 ± 5.69	14.78 ± 5.03	13.51 ± 4.87	1.72, 0.180, 0.005		
Self-report questionnaires measures										
Physical Activity Levels										
PAQ-C (points)	2.69 ± 0.53	2.55 ± 0.52	3.56	<0.001	2.64 ± 0.53	2.61 ± 0.52	2.50 ± 0.51	2.99, 0.05, 0.01		1 > 3 1 = 2 2 = 3
Sedentary Habits	Boys %	Girls %	Boys vs. Girls		Normal weight %	Overweight %	Obesity %	NW vs. OW vs. OB		
			χ^2^	*p*				χ^2^	*p*	
Inactivity during the week										
0 to 2 (hours per day)	75.7	80.40	4.63	0.099	80	80.03	65.6			
2 to 4 (hours per day)	22.2	19.1			19	18	32.3	10.23	0.04	
≥5 (hours per day)	2.1	0.5			1	1.7	2.2			
Inactivity during the weekend										
0 to 2 (hours per day)	60.7	62.8	0.314	0.855	63	66.4	49.5			
2 to 4 (hours per day)	35.6	33.9			34.1	31.1	42.9	10.43	0.03	
≥5 (hours per day)	3.6	3.3			2.9	2.5	7.7			
	Boys	Girls	Boys vs. Girls		Normal weight	Overweight	Obesity	NW vs. OW vs. OB		
	Mn ± Sd	Mn ± Sd	*t*	*p*	Mn ± Sd	Mn ± Sd	Mn ± Sd	F _(__1.700)_, *p,* *Partial η^2^*		
Well-Being										
Kid-Kindl TOT (points)	80.03 ± 9.12	80.07 ± 9.34	−0.046	0.963	80.31 ± 9.04	79.54 ± 9.40	79.36 ± 10.01	0.65, 0.525, 0.002		
Kid-Kindl PHY (points)	84.34 ± 14.65	84.45 ± 15.00	−0.097	0.923	84.54 ± 14.68	83.54 ± 15.42	84.81 ± 14.93	0.26, 0.769, 0.001		
Kid-Kindl EMO (points)	82.22 ±12.71	82.08 ± 12.75	−0.148	0.883	82.07 ± 12.66	83.18 ± 12.75	81.25 ± 13.07	0.64, 0.528, 0.002		
Kid-Kindl SE (points)	75.34 ± 16.97	73.32 ± 17.01	1.56	0.119	74.53 ± 16.94	74.20 ± 16.99	73.12 ± 17.53	0.27, 0.766, 0.001		
Kid-Kindl FAM (points)	78.90 ± 12.73	80.24 ± 11.82	−1.44	0.149	80.13 ± 11.61	78.74 ± 13.09	77.93 ± 14.27	1.62, 0.199, 0.005		
Kid-Kindl FRN (points)	77.98 ± 13.81	78.63 ± 13.25	−0.633	0.527	78.74 ± 12.94	77.68 ± 14.66	76.95 ± 14.86	0.85, 0.427, 0.002		
Kid-Kindl SC (points)	81.78 ± 15.57	82.47 ± 16.05	−0.578	0.563	82.55 ± 15.32	80.35 ± 17.55	82.35 ± 15.97	0.96, 0.384, 0.003		

NW = Normal weight; OW = Overweight; OB = Obesity; TOT = Total; BMI = Body Mass Index; WHtR = waist-to-height ratio; PAQ-C = Physical Activity Questionnaire for Children; PHY = Physical; EMO = Emotional; SE = Self Esteem; FAM = Family; FRN = Friend; SC = School. *p* < 0.05; indicates significant differences; Partial η^2^: 0.01 = small; 0.06 = medium; 0.14 = large effects size. F = variance of the group means (Mean Square Between)/mean of the within group variances (Mean Squared Error).

**Table 2:** Repeat Measure Multivariate Analysis of Variance to analyze differences in all variables between T0 and T1.

	T0	T1	Time T0 vs. T1			Time *Gender			Time * BMI Category		
	Mn ± Sd	Mn ± Sd	F _(1.700)_	*p*	*Partial η^2^*	F _(1.700)_	*p*	*Partial η^2^*	F _(2.700)_	*p*	*Partial η^2^*
Anthropometric measures											
BMI (kg/m^2^)	16.46 ± 2.35	16.54 ± 2.37	3.32	0.069	0.005	0.388	0.534	0.001	5.65	0.004	0.02
WC (cm)	57.88 ± 6.11	58.11 ± 6.08	3.31	0.069	0.005	0.075	0.784	0.001	3.73	0.024	0.01
WHtR (cm/cm)	0.48 ± 0.05	0.47 ± 0.04	43.95	<0.001	0.059	0.124	0.725	0.001	4.09	0.017	0.01
Physical Performance Measures											
SPEED (s)	8.08 ± 1.13	7.56 ± 1.04	331.82	<0.001	0.322	1.46	0.228	0.002	1.65	0.192	0.005
FLEXIBILITY (cm)	0.72 ± 6.45	2.36 ± 6.22	58.92	<0.001	0.078	3.42	0.065	0.005	0.05	0.947	0.000
STRENGTH (cm)	14.48 ± 5.48	15.43 ± 5.39	25.10	<.001	0.035	0.485	0.486	0.001	0.65	0.520	0.001
Physical Activity Levels											
PAQ-C (points)	2.62 ± 0.55	2.76 ± 0.56	52.00	<0.001	0.069	0.578	0.447	0.001	0.23	0.797	0.001
Well-Being											
Kid-Kindl TOT (points)	80.05 ± 9.23	80.92 ± 8.62	3.22	0.073	0.005	0.891	0.345	0.001	0.24	0.789	0.001
Kid-Kindl PHY (points)	84.40 ± 14.83	86.06 ± 13.80	4.48	0.028	0.010	0.658	0.417	0.001	0.01	0.998	0.000
Kid-Kindl EMO (points)	82.15 ± 12.72	83.62 ±11.64	0.4.90	0.028	0.010	0.149	0.700	0.001	0.61	0.545	0.002
Kid-Kindl SE (points)	74.29 ± 17.01	74.07 ± 16.32	0.090	0.764	0.000	2.26	0.133	0.003	0.58	0.559	0.002
Kid-Kindl FAM (points)	79.60 ± 12.27	79.35 ± 12.02	0.180	0.671	0.000	0.781	0.377	0.001	1.08	0.341	0.003
Kid-Kindl FRN (points)	78.32 ± 13.52	80.62 ± 12.06	11.28	0.001	0.016	0.020	0.887	0.001	0.24	0.783	0.001
Kid-Kindl SC (points)	82.14 ± 15.82	81.97 ± 14.64	0.051	0.821	0.000	0.243	0.622	0.001	0.49	0.614	0.001

TOT = Total; BMI = Body Mass Index; WHtR = waist-to-height ratio; PAQ-C = Physical Activity Questionnaire for Children; PHY = Physical; EMO = Emotional; SE = Self Esteem; FAM = Family; FRN = Friend; SC = School. *p* < 0.05; indicates significant differences. Partial η^2^: 0.01 = small; 0.06 = medium; 0.14 = large effects size. F = variance of the group means (Mean Square Between)/mean of the within group variances (Mean Squared Error).

**Table 3:** Chi-square to analyze difference in the sedentary behavior between T0 and T1 in Total Sample a separately for BMI categories.

Sedentary Habits Inactivity during the Week	T0 %	T1 %	T0 vs. T1		Inactivity during the Weekend	T0 %	T1 %	T0 vs. T1	
			χ^2^	*p*				χ^2^	*p*
Total Sample									
0 to 2 (hours per day)	78.2	78.9	117.02	<0.001	0 to 2 (hours per day)	61.6	65.7		
2 to 4 (hours per day)	20.5	19.1			2 to 4 (hours per day)	34.9	31.8	153.87	<0.001
≥5 (hours per day)	1.3	2.0			≥5 (hours per day)	3.5	2.7		
NW Category									
0 to 2 (hours per day)	80	80.2	76.98	<0.001	0 to 2 (hours per day)	63	67.7		
2 to 4 (hours per day)	19	17.8			2 to 4 (hours per day)	34.1	30.6	80.11	<0.001
≥5 (hours per day)	1	2.1			≥5 (hours per day)	2.9	1.7		
OW Category									
0 to 2 (hours per day)	80.3	87	19.67	<0.001	0 to 2 (hours per day)	66.4	65		
2 to 4 (hours per day)	18	12.2			2 to 4 (hours per day)	31.1	32.5	23.07	<0.001
≥5 (hours per day)	1.6	0.8			≥5 (hours per day)	2.5	2.5		
OB Category									
0 to 2 (hours per day)	65.6	62	31.46	<0.001	0 to 2 (hours per day)	49.5	54.8		
2 to 4 (hours per day)	32.3	34.8			2 to 4 (hours per day)	42.9	36.6	37.07	<0.001
≥5 (hours per day)	2.2	3.3			≥5 (hours per day)	7.7	8.6		

Note: NW = Normal weight; OW = Overweight; OB = Obesity; *p* < 0.05; indicates significant differences.

## Supplementary Materials

The following are available online at https://www.mdpi.com/1660-4601/17/17/6067/s1, Figure S1: “General form” of Self-report questionnaires measures proposed.
