# Supplementary material for: Physical Activity Habits and Well-Being among 6-Year-Old Children: The “Improving Umbrian Kids’ Healthy Lifestyle”, an Uncontrolled Pilot Study Project
Source: Int J Environ Res Public Health. 2020 Aug 20;17(17):6067. doi: 10.3390/ijerph17176067 (PMC7503798; doi:10.3390/ijerph17176067)
Supplement: Supplementary file 1 [file ijerph-17-06067-s001.pdf]

Supplementary Materials:

Figure S1: "General form" of Self-report questionnaires measures proposed

RISERVATO AL TECNICO
NOME E COGNOME DEL BAMBINO ________________
SCUOLA ____ ID _________

SEZIONE I - PER IL GENITORE

In questa scheda verranno richiesti una sola volta alcuni dati relativi al suo nucleo familiare. E' indifferente quale dei due genitori compili questa parte. *Legga con attenzione ogni domanda e metta una crocetta sulla risposta corretta.*

QUESTIONARIO COMPILATO DA: 1. Mamma del bambino 2. Papà del bambino

GENERE DEL BAMBINO: 1. M 2. F

ANNO DI NASCITA DEL BAMBINO: 1. 2006
2. 2007
3. 2008
4. 2009
5. 2010

IL BAMBINO E' NATO: 1. Italia
2. Estero

LUOGO DI NASCITA

MADRE: 1. Italia
2. Europa dell'Est
3. Europa Occidentale
4. Africa
5. Asia
6. America del Nord
7. America Latina
8. Altro __________________

PADRE: 1. Italia
2. Europa dell'Est
3. Europa Occidentale
4. Africa
5. Asia
6. America del Nord
7. America Latina
8. Altro __________________

STATO CIVILE DEI GENITORI:
1. Nubile/celibe 2. Sposati/convivente 3. Separati/divorziati 4.Vedova/o

MADRE: Età: ……… Altezza: ……… Peso attuale: ………

TITOLO DI STUDIO:
1. Scuola Elementare
2. Scuola Media Inferiore
3. 2/3 anni di Scuole Superiori
4. Scuola Media Superiore
5. Qualche anno di Università, Lauree brevi, Diploma post-maturità
6. Lauree
7. Corsi/Specializzazione Post-Università

OCCUPAZIONE:
1. Disoccupati, studenti universitari, lavori saltuari, casalinghe, pensionati
2. Lavori domestici (colf, badante, baby-sitter, bracciante)
3. Operai generici
4. Operai semi-specializzati, operatori di macchinari
5. Piccoli commercianti, artigiani, operai specializzati, affittuari agrari
6. Impiegati, geometri, ragionieri, educatori, insegnanti
7. Tecnici, semiprofessionisti, proprietari di piccole imprese
8. Professionisti minori, artisti, professionisti dello spettacolo
9. Funzionari amministrativi in grandi aziende, funzionari effettivi dell'esercito
10. Incarichi dirigenziali di alto livello, alti gradi dell'esercito, incarichi istituzionali a livello statale – regionale – provinciale

PADRE: Età: ……… Altezza: ……… Peso attuale: ………

TITOLO DI STUDIO:
1. Scuola Elementare
2. Scuola Media Inferiore
3. 2/3 anni di Scuole Superiori
4. Scuola Media Superiore
5. Qualche anno di Università, Lauree brevi, Diploma post-maturità
6. Lauree
7. Corsi/Specializzazione post-Università

OCCUPAZIONE:
1. Disoccupati, studenti universitari, lavori saltuari, casalinghe, pensionati
2. Lavori domestici (colf, badante, baby-sitter, bracciante)
3. Operai generici
4. Operai semi-specializzati, operatori di macchinari
5. Piccoli commercianti, artigiani, operai specializzati, affittuari agrari
6. Impiegati, geometri, ragionieri, educatori, insegnanti
7. Tecnici, semiprofessionisti, proprietari di piccole imprese
8. Professionisti minori, artisti, professionisti dello spettacolo
9. Funzionari amministrativi in grandi aziende, funzionari effettivi dell'esercito
10. Incarichi dirigenziali di alto livello, alti gradi dell'esercito, incarichi istituzionali a livello statale – regionale – provinciale

CI SONO ALTRI FIGLI? 1. SI 2. NO

Se SI, indicare: 1) Genere: 1. M 2. F Età: ………… Altezza: …………Peso: …………
2) Genere: 1. M 2. F Età: ………… Altezza: ………… Peso: …………
3) Genere: 1. M 2. F Età: ………… Altezza: ………… Peso: …………
4) Genere: 1. M 2. F Età: ………… Altezza: ………… Peso: …………

ORA LE FAREMO ALCUNE DOMANDE CHE RIGUARDANO IL SUO BAMBINO/A

Il suo bambino/a ha una qualche disabilità? 1. SI 2. NO

Se SI, di quale tipo? (specificare) ____

Durante il mese passato…

Mediamente, quante ore per notte ha dormito **suo figlio** durante la settimana?
1. Più di 11 ore per notte
2. Tra le 9 e le 11 ore per notte
3. Meno di 9 ore per notte

Quante ore esattamente? …………

Mediamente, quante ore per notte ha dormito **suo figlio** durante il fine settimana?
1. Più di 11 ore per notte
2. Tra le 9 e le 11 ore per notte
3. Meno di 9 ore per notte

Quante ore esattamente? …………

Come descriverebbe la qualità del sonno di suo figlio:
1. Molto buona 2. Piuttosto buona 3. Piuttosto scarsa 4. Molto scarsa

Tendenzialmente consumate almeno un pasto al giorno tutti insieme? 1. SI 2. NO

Quanti pasti consumate tutti insieme durante la settimana? 1. Meno di 4 pasti 2. 4 o più pasti

Mediamente quante ore al giorno vostro figlio passa davanti alla televisione o computer o giocando con i videogame durante la settimana**?**
1. Da 0 a 2 ore al giorno
2. Da 3 a 4 ore al giorno
3. Più di 5 ore al giorno

Mediamente quante ore al giorno vostro figlio passa davanti alla televisione o computer o giocando con i videogame durante il fine settimana**?**
1. Da 0 a 2 ore al giorno
2. Da 3 a 4 ore al giorno
3. Più di 5 ore al giorno
